# Feasibility, double-blind, randomised, placebo-controlled, multi-centre trial of hand-held NB-UVB phototherapy for the treatment of vitiligo at home (HI-Light trial: Home Intervention of Light therapy)

**DOI:** 10.1186/1745-6215-15-51

**Published:** 2014-02-08

**Authors:** Viktoria Eleftheriadou, Kim Thomas, Jane Ravenscroft, Maxine Whitton, Jonathan Batchelor, Hywel Williams

**Affiliations:** 1 Centre of Evidence Based Dermatology, University of Nottingham, Kings Meadow Campus, Lenton Lane, Nottingham NG7 2NR, UK; 2 Queens Medical Centre, Nottingham University Hospitals NHS Trust, Derby Road, Nottingham NG7 2UH, UK

**Keywords:** Hand-held phototherapy, Home phototherapy, Patient education, Randomised trial, Vitiligo

## Abstract

**Background:**

Hand-held NB-UVB units are lightweight devices that may overcome the need to treat vitiligo in hospital-based phototherapy cabinets, allowing early treatment at home that may enhance the likelihood of successful repigmentation. The pilot Hi-Light trial examined the feasibility of conducting a large multi-centre randomised controlled trial (RCT) on the use of such devices by exploring recruitment, adherence, acceptability, and patient education.

**Methods:**

This was a feasibility, double-blind, multi-centre, parallel group randomised placebo-controlled trial of hand-held NB-UVB phototherapy for the treatment of vitiligo at home. The overall duration of the trial was seven months; three months recruitment and four months treatment. Participants were randomly allocated to active or placebo groups (2:1 ratio). The primary outcome measure was the proportion of eligible participants who were willing to be randomised. The secondary outcomes included proportion of participants expressing interest in the trial and fulfilling eligibility criteria, withdrawal rates and missing data, proportion of participants adhering to and satisfied with the treatment, and incidence of NB-UVB short-term adverse events.

**Results:**

Eighty-three percent (45/54) of vitiligo patients who expressed interest in the trial were willing to be randomised. Due to time and financial constraints, only 29/45 potential participants were booked to attend a baseline hospital visit. All 29 (100%) potential participants were confirmed as being eligible and were subsequently randomised. Willingness to participate in the study for General Practice (family physicians) surgeries and hospitals were 40% and 79%, respectively; 86% (25/29) of patients adhered to the treatment and 65% (7/11) of patients in the active group had some degree of repigmentation. Only one patient in the active group reported erythema grade 3 (3%). Both devices (Dermfix 1000 NB-UVB and Waldmann NB-UVB 109) were acceptable to participants.

**Conclusions:**

Hand-held NB-UVB devices need evaluation in a large, pragmatic RCT. This pilot trial has explored many of the uncertainties that need to be overcome before embarking on a full scale trial, including the development of a comprehensive training package and treatment protocol. The study has shown strong willingness of participants to be randomised, very good treatment adherence and repigmentation rates, and provided evidence of feasibility for a definitive trial.

**Trial registration:**

NCT01478945

## Background

Vitiligo is the most common depigmentation disorder of the skin affecting around 0.5% of the population worldwide. The British Association of Dermatologists clinical guidelines for the management of vitiligo recommend narrow-band ultraviolet light B (NB-UVB) (311 to 312 nm), tacrolimus, and topical steroids to treat the condition [1]. The Cochrane systematic review update concluded that light combination interventions were superior to monotherapies [2]. However, larger studies are needed to provide stronger evidence for the many combination interventions that have shown promise in treating vitiligo [2].

Evidence suggests that early treatment of vitiligo may increase the possibility of successful repigmentation even on the most resistant areas such as hands. In early stages of vitiligo, some functioning melanocytes are still present, providing a possible explanation of these results [3-5]. Recently, a new European Guideline for vitiligo was published, suggesting early treatment of small lesions of recent onset and childhood vitiligo with combination of phototherapy and topical agents [6]. However, suitable facilities and equipment are needed if this new guideline is to be implemented.

### Phototherapy for the treatment of vitiligo

NB-UVB is available in secondary care, requires regular hospital visits, and usually involves whole body cabinets suitable for extensive vitiligo [1]. Currently, there are various devices available on the market for the delivery of NB-UVB: whole body units, hand and feet units, and hand-held units. The choice of devices is usually based on the size and location of the lesions and the percentage of the body surface affected [7]. Hand-held NB-UVB units are portable and light weight devices, suitable for the treatment of small areas of skin. Benefits of using hand-held devices at home include the reduction in number of hospital visits, sparing of uninvolved skin, fewer costs for patients (such as travelling costs), and the ability to treat at an early stage of the disease when the intervention might be more effective [8].

Although there are currently no studies evaluating hand-held NB-UVB devices for vitiligo, participants have reported benefit [9]. Further, trials using these devices for home treatment of scalp psoriasis have shown that they are effective, well tolerated, easy to use, and safe [10,11].

The effectiveness and safety of NB-UVB for the treatment of vitiligo has been identified to be an important research topic for both patients and clinicians [12]. Feedback from patients (via the Vitiligo Society UK and Vitiligo Support International) suggests that patients are currently buying hand-held NB-UVB units and using them at home unsupervised. Moreover, if hand-held devices prove to be effective and safe for the treatment of vitiligo at home, this could be an important addition to the treatment options available to patients with focal or early vitiligo.

### Objectives

The main aim of this pilot trial was to determine the feasibility of conducting a national, multi-centre, randomised controlled trial (RCT) to determine the effectiveness and safety of home hand-held NB-UVB phototherapy units both compared to and in combination with topical treatments for repigmentation of early and/or focal vitiligo. The primary objective of this pilot trial was to establish the proportion of eligible patients with vitiligo who are willing to be randomised to home NB-UVB (number of randomised participants/number of eligible participants at baseline). In this trial, two different hand-held NB-UVB devices, Dermfix 1000 NB-UVB and Waldmann NB-UVB 109, with the same output, but differing with regards to the size of treatment area, weight of the unit, cable length, and price were tested. The rationale behind using two different devices was to monitor and assess which of the two units was best tolerated in terms of participant satisfaction. The information gathered assisted in the choice of device for the main RCT.

An innovative training package was developed for participants explaining how to use the intervention and how to deal with side effects. The successfulness of this training package was established by measuring the following secondary objectives: i) preparation of a training package for participants explaining how to use the intervention and how to deal with possible side effects; ii) estimation of withdrawal rates and missing data; iii) establishment of participants’ adherence to and satisfaction with the treatment; and iv) establishment of the occurrence of possible short-term side effects, i.e., if the device is suitable for home use with limited medical supervision.

Another secondary objective of this pilot trial was the testing of feasibility outcomes for the main RCT including repigmentation, cessation of spreading of the disease, impact on quality of life, global improvement in vitiligo, patient’s benefit index, and colour match.

## Methods

### Ethics approval

This trial was approved by the National Research Ethics Service committee of East Midlands (REC reference: 11/EM/0331) and was registered with clinicaltrials.gov (ISRCTN: NCT01478945).

### Trial configuration

This was a feasibility, double-blind, multi-centre, parallel group randomised placebo-controlled trial of hand-held NB-UVB phototherapy for the treatment of vitiligo at home. The acronym for this trial was *HI-Light trial* for vitiligo (Home Intervention of Light therapy trial for vitiligo). The overall duration of the trial was seven months: a three-month recruitment period (1st of March 2012 to 31st of May 2012) and a four-month treatment period (until 31st of September 2012).

Participants were recruited at the Queen’s Medical Centre and NHS Treatment Centre in Nottingham, and at Leicester Royal Infirmary in Leicester. Primary Care Research Networks in both Leicester and Nottingham were involved. King’s Mill Hospital in Mansfield and local GP practices (Nottingham and Leicester) were used as participant identification centres along with direct advertising to participants through the Vitiligo Society UK and the Centre of Evidence Based Dermatology.

### Randomisation and blinding

Participants were randomly allocated to an active or placebo group in a 2:1 ratio. Participants in the active group were allocated to two different hand-held devices, Dermfix 1000™ and Waldmann™, in a 1:1 ratio. The randomisation was based on a computer generated pseudo-random code, using random permuted blocks of randomly varying size, created by the Nottingham Clinical Trials Unit in accordance with their standard operating procedure and held on a secure University of Nottingham server. The randomisation was stratified by the recruiting site (Nottingham and Leicester). Participants, research nurses, dermatologists, and independent outcome assessors were blinded. Only the trial administrator and the data manager at the Nottingham Clinical Trials Unit were aware of participants’ allocation.

### Participants and settings

The principal investigators of both recruiting centres compiled a list of prospective vitiligo patients (September 2011 to January 2012). Patient identification centres ran searches in their databases using the following criteria: diagnosis of vitiligo and age of 5 years old or older. A dedicated website (http://www.vitiligostudy.org.uk) was available for the purpose of this trial. Participants with non-segmental, spreading or stable vitiligo (confirmed by a dermatologist), affecting <25% of their body surface area, older than 5 years old, were included in the trial. No therapy for vitiligo in the previous two weeks and no other concurrent vitiligo treatments during the trial were allowed. For each participant, up to three vitiliginous lesions were chosen, preferably on three different anatomical areas. Exclusion criteria were: segmental or universal vitiligo, previous history of skin cancer, recent/concurrent radiotherapy, photosensitivity, use of immunosuppressive or photosensitive drugs, pregnant or lactating women, major medical co-morbidities, and vitiligo limited to the genitalia only.

All participants (or their parent/legal guardian if a participant was under the age of 16) provided written informed consent before they entered the trial. In addition, a second consent form was signed on completion of the NB-UVB training session.

### Outcomes

The primary outcome measure for this pilot trial was the proportion of eligible participants who were willing to be randomised (number of randomised participants/number of eligible participants at baseline).

The secondary outcomes were:

● Proportion of participants expressing interest in the trial (number of participants pre-screened/number of invitation sent) and fulfilling eligibility criteria (number of participants eligible at baseline visit/number of participants pre-screened).

● Withdrawal rates and missing data.

● Proportion of participants adhering to and satisfied with the treatment (number of participants who complied with the treatment regimen/total number of randomised participants). Adherence was monitored using all of the following parameters:

○ Three to four treatment sessions per week.

○ At least one day should be left between consecutive treatment sessions.

○ If a treatment session was missed due to side effects and the treatment plan was resumed correctly, participants were considered compliant with the treatment plan.

● Incidence of NB-UVB short-term adverse events (erythema (Grade 1–4), pruritus, perilesional hyperpigmentation, hypersensitivity reactions, cold sores, dry skin).

● Proportion of participants and assessors for whom the blinding of the allocated group (active/placebo) was maintained.

Outcome measures for the main large trial were also tested:

● Repigmentation rate of vitiliginous lesions presented in percentage of repigmentation quartiles: negative–0%, 1–24%, 25–49%, 50–74%, 75–100%. ConvaTec transparencies were used to trace the lesions at baseline and week 16 visits. These were measured by using the ImageJ 1.47d (Image processing and analysis in Java by the National Institute of Health, USA; http://imagej.nih.gov/ij).

● Cessation of spreading of vitiligo during the past year, i.e., no new vitiliginous lesions or no increase in size of existing vitiliginous lesions in the last 12 months.

● Impact on the quality of life of participants: Dermatology Life Quality Index (DLQI) [13] and Children Dermatology Life Quality Index (CDLQI) [14] on baseline and week 16 visits.

● Global improvement in vitiligo: 5-point Likert scale (much worse; a bit worse; no change; a bit better; much better) at week 16 visit.

● Patient Benefit Index (PBI) [15] at baseline and week 16 visit.

● Colour match of newly repigmented vitiliginous lesions (bad, fair, or excellent). Patient and research nurse were asked to rate the colour match of each representative lesion at the week 16 visit. This outcome was subjective.

### Trial procedures

**Figure 1:**
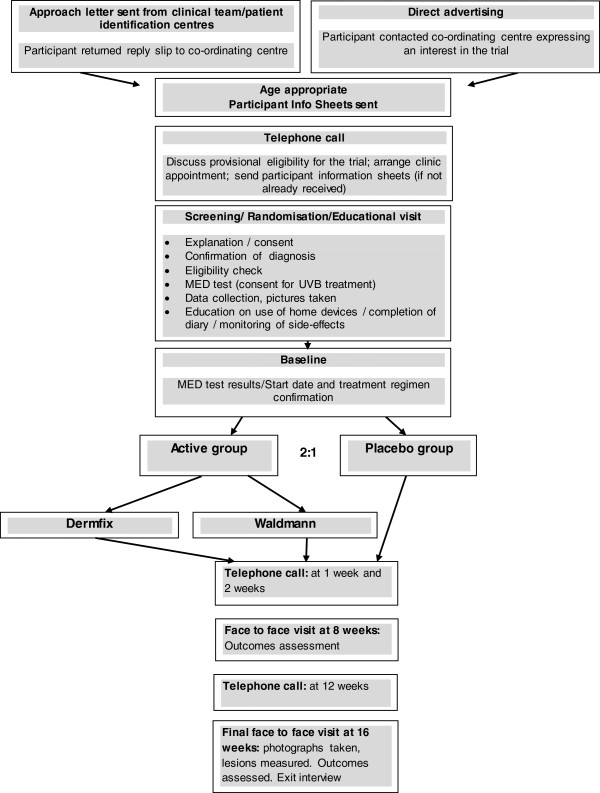
Flowchart of HI-Light trial configuration.

On receipt of a potential participant’s contact details, an age-appropriate information sheet was sent and a member of the research team made contact. Participants were pre-screened over the telephone. A hospital visit for successfully pre-screened patients was booked. The initial trial visit took approximately 1.5 to 2 hours and included a training session on how to complete the treatment diary and adjust treatment time accordingly to the erythema response and what to do in case of short-term side effects. Participants returned to the hospital the following day and their Minimal Erythema Dose (MED) results were read. Participants had to return to the hospital at week 8 and week 16 (final face-to-face visit) (Figure 1).

### Intervention

In the active group, two different hand-held NB-UVB devices were explored: Dermfix 1000™ NB-UVB and Waldmann™ NB-UVB 109. The placebo device was identical to the active device Dermfix 1000, with the only difference being that a special plastic cover blocked the emission of NB-UVB rays. The output of each of the hand-held devices was tested before and after the trial.

### Treatment plan and adherence

The treatment was self-administered by the participants or their carers on alternate days, i.e., three to four times a week but never on consecutive days. Each participant received a personalised treatment plan according to his/her Fitzpatrick skin type: I, II, III, and IV–VI (Table 1). In addition, the results of the MED test-determined skin type were compared to the dermatologist recorded skin type. This was important in order to determine whether or not the MED test was necessary for the future RCT, or whether the treatment plan based on a dermatologist recorded skin type alone was safe.

**Table 1:** Summary of treatment schedules according to the participants’ skin type

**Skin type**	**Starting time**	**Exposure time: +20% of treatment 1**	**Exposure time: −20% of treatment 1**	**Maximum exposure time (MET)**	**Total duration**
I	15 sec	+3 sec	−3 sec	3 min	4 months
II	20 sec	+4 sec	−4 sec	4 min	4 months
III	25 sec	+5 sec	−5 sec	5 min	4 months
IV	30 sec	+6 sec	−6 sec	6 min	4 months
V	30 sec	+6 sec	−6 sec	6 min	4 months
VI	30 sec	+6 sec	−6 sec	6 min	4 months

Participants’ adherence to the treatment was monitored by reviewing their diaries. The number of treatment sessions per week and treatment times were used as parameters to monitor participants’ compliance.

### Statistical analysis

Demographic characteristics of the participants, measures of adherence to the treatment plan, and all other outcomes data, including outcomes for the main RCT, were summarised by descriptive statistics or frequency tables, stratified by active/placebo groups. No formal statistical analyses were performed on outcome measures since this was a pilot study to determine feasibility of a definitive trial. All analyses were performed using Stata SE 11 and MS Excel 2007.

### Sample size

This was a pilot study, with sample size being resource-driven in terms of available participants in a reasonable time-frame, for which no formal statistically based sample size estimate was applicable. A minimum of 21 participants from two recruitment sites was deemed appropriate to compare the devices and to measure recruitment rates for each site.

## Results

### Recruitment and eligibility

**Figure 2:**
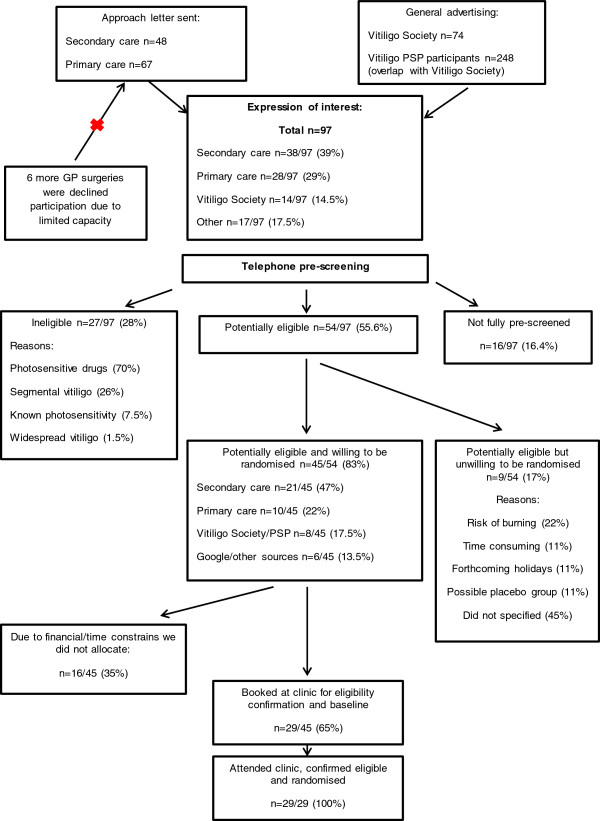
Summary of recruitment sources into the HI-Light trial for vitiligo.

In total, 97 people approached the research team, expressing an interest in this pilot trial (Figure 2). Forty-eight invitation letters were sent to vitiligo patients who attended dermatology departments at the Queen’s Medical Centre/NHS Treatment Centre in Nottingham (31 patients) and the Leicester Royal Infirmary (17 patients). We received 38 (response rate = 79% (38/48)) completed reply slips from patients, who were willing to be contacted (Nottingham response rate = 93.5% (29/31); Leicester response rate = 53% (9/17)). In addition, two GP surgeries (one in Leicester and one in Nottingham) sent 67 invitation letters. From these, we received 28 completed reply slips (total response rate = 40% (28/67)). In addition, six more GP practices expressed an interest in taking part in this trial. However, due to the overwhelming response from the secondary care patients and lack of time and resources for further recruitment, these surgeries were asked not to send invitation letters. In addition, the Vitiligo Society UK sent 74 invitation letters to its members living in Nottingham, Leicester, and Birmingham. Fourteen patients were interested in the trial (response rate = 19% (14/74)). Finally, patients held on a mailing list at the Centre of Evidence Based Dermatology were also informed about the trial (n = 284).

In total, 55.6% (54/97) of people who expressed interest in the trial were successfully pre-screened by telephone and provisionally met the trial eligibility criteria; 83% of them (45/54) were willing to attend the baseline visit and be randomised (Figure 2).

**Figure 3:**
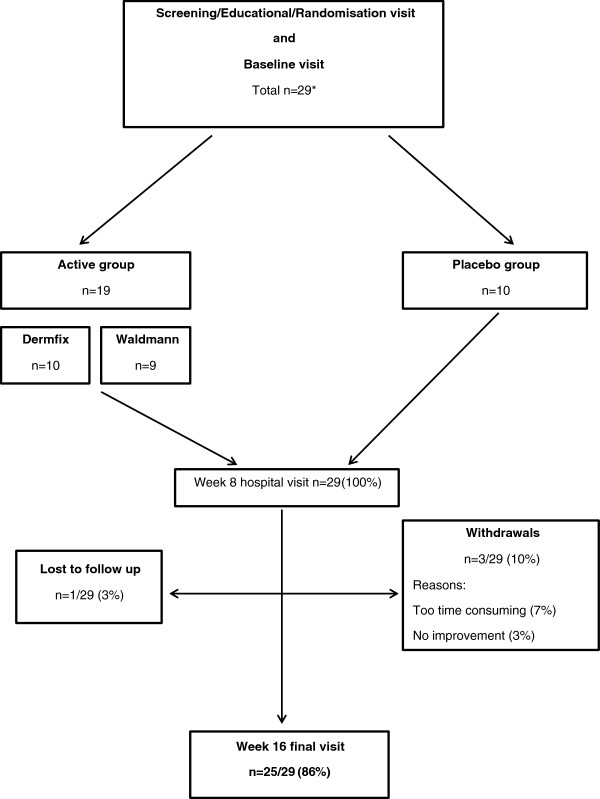
Flow-diagram of randomised participants into the HI-Light trial.

Due to time and financial constraints, only 29 of 45 potential participants were booked to attend a baseline hospital visit on a “first come-first served” basis. All 29 potential participants who attended the baseline visit were confirmed as being eligible and were subsequently randomised into the trial (29/29; 100%) (Figure 3).

In total, 29 participants with 84 representative lesions were randomised into the two groups (Table 2) at a ratio of 2:1 (active: placebo). Within the active group, patients were randomised to two different active devices (Figure 3).

**Table 2:** Baseline characteristics of participants of HI-Light trial

**Baseline characteristics**	**All groups**	**Active group**	**Placebo group**
Number of participants	29	19	10
Age range (years)	5–71	5–71	13–51
Adult/child	23/6	14/5	9/1
Age mean (years); SD	31.7 ± 17.9	27.63 ± 18.6	39.4 ± 13.5
Adults	38.6 ± 14.8	38 ± 15.8	42.3 ± 10.85
Children	10.25 ± 3.5	9.9 ± 3.6	13 ± n/a
Sex (female/male)	15/14	10/9	5/5
Ethnicity:			
White British	20/29 (69%)	12/19 (64%)	8/10 (80%)
Mixed	1/29 (3.5%)	1/19 (5%)	None
Black/Black Caribbean	2/29 (7%)	2/19 (11%)	None
Indian	2/29 (7%)	1/19 (5%)	1/10 (10%)
Pakistani	1/29 (3.5%)	1/19 (5%)	None
Asian	2/29 (7%)	1/19 (5%)	1/10 (10%)
Other ethnic group	1/29 (3.5%)	1/19 (5%)	None
Duration of vitiligo (years)			
Mean ± SD (min; max)	12.28 ± 9.67 (min = 2; max = 33)	11.36 ± 10.12 (min = 2; max = 33)	14.01 ± 8.5 (min = 5; max = 28)
Vitiligo activity per participant (overall)			
Stable	6	3	3
Spreading	19	14	5
Repigmenting	4	2	2
BSA% covered by vitiligo Mean ± SD (min; max)	8.83 ± 6.19 (min = 2; max = 25)	9.84 ± 5.96 (min = 3; max = 25)	6.9 ± 6.17 (min = 2; max = 21)
Number of lesions	84	56	28

### Withdrawals

Three of 29 participants (10%) withdrew from the treatment (two patients from the active group and one from placebo group). The reasons for withdrawals were that the treatment was too time consuming (3%; 1/29) and the lack of improvement in vitiligo (7%; 2/29). Only one (3%) participant was lost to follow-up (Figure 3).

### Missing data

All participants, except one who was lost to follow-up, completed the end of study questionnaire in full (28/29; 96.5%). The DLQI and PBI questionnaires at baseline and week 16 were completed by 96.5% (28/29) of participants also; at baseline one participant did not complete the questionnaires. One missing diary and week 16 questionnaire (3%; 1/29) belonged to the patient lost to follow-up. The research team made every effort possible to contact and find the patient, including reaching out to his regular GP. Unfortunately, neither the diary nor the device were recovered.

### Adherence

All but one treatment diary (28/29; 97%) were returned and analysed; 86% of participants (25/29) completed the four-month treatment course, of which 84% (21/25) administered phototherapy at home three to four times a week and reduced treatment time correctly. Common mistakes were: no treatment time reduction (6/25; 24%) or incorrect time reduction (1/25; 4%) following a missed treatment session. These minor deviations from the treatment schedule did not result in serious side effects.

### Satisfaction with the treatment

Participants in the active group were more likely to be satisfied with their treatment than participants allocated to the placebo group (31.5% versus 20%). The majority of participants in both groups said they would use the hand-held device again and recommend it to others (68% (19/28) and 64% (18/28), respectively).

Both active devices (Dermfix™ and Waldmann™) received the following positive comments: easy to use, portable, compact, convenient to operate at home, no need of coming to the hospital, easy to perform, and flexible. Additional positive comments on the Dermfix devices related to its convenient shape.

### Side effects

Erythema grade 1 and 2 was reported in 27% (8/29) and 13% (4/29) of participants, respectively, in the active group. Only one patient reported erythema grade 3 (3%). Other side effects included: pruritus (7% (2/29)), hyperpigmentation around the lesions (10% (3/29)) and dry skin (10% (3/29)), cold sores (3% (1/29)). In the placebo group, two patients reported erythema grade 1 (6% (2/29)). No other side effects were reported in the placebo group.

### Repigmentation

At baseline, the mean size of lesions in the active and placebo groups were 15.42 cm^2^ (SD: ± 20.2 cm^2^) and 20.54 cm^2^ (SD: ± 19.4 cm^2^), respectively. At the week 16 hospital visit, the mean size of lesions in the active and placebo groups were 14.43 cm^2^ (SD: ± 18 cm^2^) and 21 cm^2^ (SD: ± 20.85 cm^2^), respectively.

High grade repigmentation (75–100%) was noted in 12% (1/17) of participants in the active group, compared to none in the placebo group. In the active group, 75% (29/39) of all lesions showed some repigmentation compared to 39% of lesions in the placebo group. Overall, the anatomical sites which responded best to treatment were the face and neck (Tables 3, 4, 5).

**Table 3:** Mean percentage of repigmentation after 16 weeks of treatment with home phototherapy per participant

**Number of participants in:**	**Mean % repigmentation after 16 weeks of treatment per participant**					
	**Negative–0**	**1–24**	**25–49**	**50–74**	**75–100**	**Total**
Active group	6 (35%)	8 (47%)	1 (6%)	0	2 (12%)	17
Placebo group	4 (40%)	6 (60%)	0	0	0	10
Total	10 (37%)	14(52%)	1 (4%)	0	2 (7%)	27

When analysing the overall treatment response per participant, mean percentage of repigmentation of all representative lesions for each participant was estimated. This was done by simply finding the total difference in size, of all lesions at baseline and week 16 in cm^2^. The results were afterwards converted into percentages and allocated to one of the five groups (negative–0%, 1–24%, 25–49%, 50–74%, 75–100%).

**Table 4:** Repigmentation in the active group for each anatomical site per participant

	**Anatomical site**				
	**Face/neck**	**Trunk**	**Upper limbs**	**Lower limbs**	**Hands/feet**
Number of participants*	6	6	13	11	3
Repigmentation:					
Negative–0%	2 (33%)	3 (50%)	3 (22%)	2 (18%)	0
1–24%	1 (17%)	2 (33%)	8 (62%)	7 (64%)	2 (67%)
25–49%	1 (17%)	0	1 (8%)	2 (18%)	1 (33%)
50–74%	0	0	0	0	0
75–100%	2 (33%)	1(17%)	1 (8%)	0	0
Baseline: mean lesion size (cm^2^ ± SD)	75.4 ± 7.4	13 ± 15.9	11.2 ± 10.9	21 ± 29.1	18.9 ± 25.7
16 week: mean lesion size (cm^2^ ± SD)	47.4 ± 4.4	13.7 ± 17.4	10.3 ± 9.6	20.6 ± 25.8	22.6 ± 26.6

*One lesion per anatomical site per participant was analysed. If two or more lesions were available on the same anatomical site, the lesion on the right side was chosen. If no lesions were available on the right side, then the lesion, which repigmented the most, was included in the analysis.

**Table 5:** Repigmentation in the placebo group for each anatomical site per participant

	**Anatomical site**				
	**Face/neck**	**Trunk**	**Upper limbs**	**Lower limbs**	**Hands/feet**
Number of participants*	5	1	8	5	4
Repigmentation:					
Negative–0%	3 (60%)	0	3 (37%)	5(100%)	2 (50%)
1–24%	1 (20%)	1(100%)	5 (63%)	0	2 (50%)
25–49%	1 (20%)	0	0	0	0
50–74%	0	0	0	0	0
75–100%	0	0	0	0	0
Baseline: mean lesion size (cm^2^ ± SD)	10.7 ± 5.6	34.8	22.6 ± 18.4	22.6 ± 24	20.9 ± 8.8
16 week: mean lesion size (cm^2^ ± SD)	11.3 ± 7	28.2	23.2 ± 21.6	26.1 ± 27.2	20.7 ± 6.4

*One lesion per anatomical site per participant was analysed. If two or more lesions were available on the same anatomical site, the lesion on the right side was chosen. If no lesions were available on the right side, then the lesion, which repigmented the most, was included in the analysis.

### Cessation of spreading of vitiligo

In the active group, 44% of lesions (22/50) remained stable throughout the trial compared to 72% (13/28) of lesions in the placebo group; 27% (11/50) and 22% (4/18) of stable lesions started repigmenting in the active and the placebo groups, respectively.

### Global improvement in vitiligo

Patients rated their vitiligo as being “much better” in 17% (3/18) of cases in the active group compared to none in the placebo group. Similarly, 23% (4/17) of research nurses and independent outcome assessors rated patients’ vitiligo as being “much better” in the active group compared to 0% in the placebo group.

### Colour match of vitiliginous patches

In the active group, around one third of newly repigmented lesions were rated as good to excellent by patients, research nurses, and independent assessors (30% (16/53), 32% (16/50), and 24% (12/50), respectively). On the other hand, no one rated newly repigmented lesions as good or excellent in the placebo group.

### Quality of life

There was a little change in the DLQI scores from baseline (active groups mean ± SD: 2.8 ± 3.2, min = 0, max = 6; placebo group mean ± SD: 3.8 ± 3.2, min = 0, max = 10) to week 16 (active groups mean ± SD: 3.2 ± 2.3, min = 0, max = 7; placebo group mean ± SD: 3.7 ± 3.8, min = 0, max = 12) in both the active and placebo groups, suggesting that the treatment had little impact on quality of life. The CDLQI was not analysed due to insufficient numbers of children in the trial.

### Benefit evaluation in vitiligo

There was no difference between the active and placebo groups in the PBI index. The mean PBI for the active group (17 participants) was 0.92 (SD: ± 1.16; min = 0; max = 3.68) and mean PBI for the placebo group (10 participants) was 0.91 (SD: ± 0.99; min = 0; max = 3.26). Both groups reported a PBI of approximately 1 (“slight benefit”).

### Success of blinding

At the end of the trial, 70% (19/27) of participants and 40% (16/27) of research nurses guessed treatment allocation correctly. The main reasons for unblinding of the research nurses were erythema (30% (3/10)) and improvement in vitiligo (60% (6/10)) in the active group, and lack of treatment response (100% (6/6)) in the placebo group.

### Minimal erythema dose

In only half of patients (55% (16/29)), the starting dose as determined by the MED test was the same as that estimated by a dermatologist based on skin type alone. In 17% (6/29) of participants, the MED test results showed that their skin was more sensitive to sunlight than determined by a dermatologist. In 24% (7/29) of participants, the MED results were higher than the ones determined by dermatologists allowing them to be prescribed a higher dose of NB-UVB.

## Discussion

The majority of clinical treatment recommendations for vitiligo are based on small, inconclusive trials, specialist consensus, and data from other skin diseases [1,2]. Perhaps unsurprisingly, patients often feel abandoned and believe that they do not receive adequate support from their doctors [16].

The HI-Light pilot trial was the first trial evaluating the safety of using hand-held phototherapy at home and testing the feasibility of conducting the first national multi-centre RCT on home hand-held phototherapy for vitiligo. Although this was not an efficacy trial, this pilot trial was a crucial preliminary step to support a grant application for a national multi-centre RCT and to develop a training package on home targeted phototherapy for vitiligo.

### Summary of the main findings

Recruitment into the trial went surprisingly well. The target number of participants was exceeded easily and the recruitment period lasted only three months instead of six.

Telephone pre-screening of potential participants prior to the hospital visit proved to be very successful. Pre-screening participants over the phone saved time and resources, considering that both a dermatologist and a research nurse had to be present during the initial hospital visit and a room had to be booked in a busy dermatology clinic for each potential participant.

Only 10% of participants withdrew from the trial compared to the previously reported 20% in a trial on hospital phototherapy [17]. The findings of the trial suggest that patients with vitiligo are very keen to take part in trials on home light phototherapy using hand-held devices. The main reason for this was the fact that the treatment was self-administered at home, allowing flexibility around days and times for administration.

The training session and materials on how to self-administer home phototherapy were comprehensive, adequate, and easy to follow, as demonstrated by only one incident of erythema grade 3 and high levels of adherence to the treatment plan (86%). Erythema grades 1 and 2 are generally considered acceptable and indicate response to light therapy. Surprisingly, two patients in the placebo group also reported erythema grade 1. The most likely explanation for this might be confusion of erythema grade 1 with erythema caused by normal warming up of the device during treatment.

Satisfaction with the treatment in both active and placebo groups only differed slightly (31.5% versus 20%). Further, active and placebo groups reported only a “slight benefit” from the treatment and, in general, did not show any changes in their quality of life. This comes as no surprise as the treatment period was clinically inadequate given the limited duration of this pilot trial. On the other hand, 75% of lesions in the active groups showed some degree of repigmentation. In the clinical setting, this is usually an indication to continue phototherapy treatment [1].

### Limitations of this trial

The main limitations of this trial were the short treatment period and the small sample size, although it was not designed to be an efficacy trial. Nevertheless, four months was adequate to capture initial treatment response, if any, and therefore provide an indication as to whether or not treatment should be continued.

Although the secondary care recruitment was excellent, this is likely to be lower in centres where there is no adequate research nurse support.

The unsuitability of the ConvaTec transparencies for skin mapping of vitiliginous lesions was a potential source of measurement error in the repigmentation measurements. As this pilot trial did not seek to answer efficacy questions on home hand-held phototherapy, the above did not affect the validity of this trial.

### Recommendations for future trials

In order to inform future trials on home hand-held phototherapy, the following recommendations are provided:

*Recommendation 1:* Recruitment through primary care (General Practice surgeries). Primary care is likely to be the main source of potential participants (especially for early and limited disease). In addition, in order to reduce the burden on clinic space and availability of investigators, it is recommended that potential participants are pre-screened over the telephone.

*Recommendation 2:* Skin mapping using transparencies. Skin mapping captures the three-dimensional character of vitiliginous lesions, does not require standardisation, specific body positioning, or expensive equipment. It is also easy to replicate and relatively cheap. On the other hand, the pattern of repigmentation (perifollicular or diffuse), and the colour match of newly repigmented lesions made it difficult to clearly identify the edges of the vitiliginous area. Based on the above, it is important to identify a suitable (very thin, flexible, and not shiny) transparency, which could also be scanned into a digital image without the need for manual transfer. Good lighting in the clinic room and contouring of the vitiliginous lesion with a surgical skin mapping pen are recommended.

*Recommendation 3:* Outcomes for the main trial. On the basis of the results of the pilot trial, the following outcomes are recommended to be included in the main trial: repigmentation, patient reported success, cessation of spreading of vitiligo, and impact on quality of life of vitiligo patients, amongst others that researchers deem appropriate for their trial. Also, based on the above recommendation (2), researchers should consider choosing patient-reported success rather than repigmentation as a primary outcome. Although the former is a subjective outcome, perhaps it better captures several aspects of repigmentation such as pattern of repigmentation, colour match of newly repigmented lesions, and percentage of repigmentation (size of vitiliginous lesions).

*Recommendation 4:* MED testing is recommended for future trials on home hand-held phototherapy and is necessary to ensure patients’ safety and appropriate NB-UVB dose administration.

*Recommendation 5:* A training DVD on how to use hand-held devices at home has been produced in order to standardise this intervention and ensure consistency in the training provided (http://www.nottingham.ac.uk/research/groups/cebd/index.aspx). The training package developed for this trial proved to be comprehensive, well understood, and safe.

*Recommendation 6:* Inclusion of active treatments in all groups would help to avoid unblinding resulting from differential treatment response. Although it is possible to mask the treatment allocation, this is likely to be compromised and should therefore be planned for.

## Conclusions

In light of newly emerging evidence that early treatment of vitiliginous lesions seems to be effective [3-5], a trial utilising hand-held phototherapy at home seems an appropriate way forward.

The HI-light pilot trial showed that the training on hand-held phototherapy was comprehensive and well tolerated. This intervention seems to be safe for the home-treatment of vitiligo in isolated areas of the body in both adults and children.

In conclusion, the results of this pilot trial strongly suggest that a national multi-centre RCT involving home hand-held phototherapy is both feasible and acceptable to patients and clinicians. It would address an important area of unmet need, potentially providing a useful treatment strategy for patients with limited/early disease and assist future research into the treatment of vitiligo, based on the topics of importance for patients and clinicians [12].

## Abbreviations

CDLQI: Children Dermatology Life Quality Index; DLQI: Dermatology Life Quality Index; HI-Light trial: Home Intervention of Light therapy trial for vitiligo; MED: Minimal Erythema Dose; NB-UVB: Narrow-band ultraviolet light B; PBI: Patient Benefit Index; RCT: Randomised controlled trial.

## Competing interests

The authors declare that they have no competing interests.

## Authors’ contribution

VE conceived the study. VE developed the training package on home hand-held phototherapy.VE and KT performed the statistical analysis. VE, KT, JR, JB, MW, and HW co-ordinated the trial from the Centre of Evidence based Dermatology. JR was Principal Investigator for Nottingham University Hospitals NHS Trust). VE, JR, and JB recruited participants for the trial. All authors participated in its design and helped to draft the manuscript. All authors read and approved the final manuscript.

## Authors’ information

Kim Thomas, Jane Ravenscroft, Maxine Whitton, Jonathan Batchelor, and Hywel Williams are co-authors.

## References

[B1] Gawkrodger DJ Ormerod AD Shaw L Mauri-Sole I Whitton ME Watts MJ Anstey AV Ingham J Young K Guideline for the diagnosis and management of vitiligo B J Dermatol 2008 159 5 1051 1076 10.1111/j.1365-2133.2008.08881.x 19036036

[B2] Whitton ME Pinart M Batchelor J Lushey C Leonardi-Bee J Gonzalez U Interventions for vitiligo Cochrane Database Syst Rev 2010 1 CD003263 20091542 10.1002/14651858.CD003263.pub4

[B3] Lee DY Kim CR Lee JH Recent onset vitiligo on acral areas treated with phototherapy: need of early treatment Photodermatol Photoimmunol Photomed 2010 26 2 266 268 21175856 10.1111/j.1600-0781.2010.00530.x

[B4] Lee DY Kim CR Lee JH Yang JM Recent onset vitiligo treated with systemic corticosteroid and topical tacrolimus: need for early treatment in vitiligo J Dermatol 2010 37 12 1057 1059 10.1111/j.1346-8138.2010.00929.x 21083711

[B5] Hallaji Z Ghiasi M Eisazadeh A Damavandi MR Evaluation of the effect of disease duration in generalized vitiligo on its clinical response to narrowband ultraviolet B phototherapy Photodermatol Photoimmunol Photomed 2012 28 3 115 119 10.1111/j.1600-0781.2012.00648.x 22548391

[B6] Taieb A Alomar A Bohm M Dell'anna ML De Pase A Eleftheriadou V Ezzedine K Gauthier Y Gawkrodger DJ Jouary T Leone G Moretti S Nieuweboer-Krobotova L Olsson MJ Parsad D Passeron T Tanew A van der Veen W van Geel N Whitton M Wolkerstorfer A Picardo M Guidelines for the Management of Vitiligo: the EDF consensus by the writing group of the Vitiligo European Task Force (VETF) in cooperation with the European Academy of Dermatology and Venereology (EADV) and the Union Europeenne des Medecins Specialistes (UEMS) B J Dermatol 2013 168 1 5 19 10.1111/j.1365-2133.2012.11197.x 22860621

[B7] Ibbotson SH Bilsland D Cox NH Dawe RS Diffey B Edwards C Farr PM Ferguson J Hart G Hawk J Lloyd J Martin C Moseley H McKenna K Rhodes LE Taylor DK An update and guidance on narrowband ultraviolet B phototherapy: a British Photodermatology Group Workshop Report B J Dermatol 2004 151 2 283 297 10.1111/j.1365-2133.2004.06128.x 15327535

[B8] Mysore V Targeted phototherapy Indian J Dermatol Venereol Leprol 2009 75 2 119 125 10.4103/0378-6323.48655 19293497

[B9] Yule S Narrow-band UVB, (TL-01) phototherapy comb (Psoracomb) for scalp psoriasis treatment Dermatolog Nurs 2005 4 23 25

[B10] Dotterud LK Braun R [UV-B comb versus betamethasone solution in scalp psoriasis] Tidsskr Nor Laegeforen 2000 120 16 1858 1859 10925612

[B11] Caccialanza M Piccinno R Cappio M Rozza M Mainardi L [Phototherapy of psoriasis of the scalp. Results in 21 patients treated with a special portable ultraviolet rays lamp] Giornale Italiano Dermatologia Venereologia 1989 124 11–12 LXI LXV 2638640

[B12] Eleftheriadou V Whitton ME Gawkrodger DJ Batchelor J Corne J Lamb B Ersser S Ravenscroft J Thomas KS Future research into the treatment of vitiligo: where should our priorities lie? Results of the vitiligo priority setting partnership B J Dermatol 2011 164 4 530 536 10.1111/j.1365-2133.2010.10160.x PMC3084501 21128908

[B13] Finlay AY Khan GK Dermatology Life Quality Index (DLQI)-a simple practical measure for routine clinical use Clin Experim Dermatol 1994 19 3 210 216 10.1111/j.1365-2230.1994.tb01167.x 8033378

[B14] Lewis-Jones MS Finlay AY The Children’s Dermatology Life Quality Index (CDLQI): initial validation and practical use B J Dermatol 1995 132 6 942 949 10.1111/j.1365-2133.1995.tb16953.x 7662573

[B15] Augustin M Gajura AI Reich C Rustenbach SJ Schaefer I Benefit evaluation in vitiligo treatment: development and validation of a patient-defined outcome questionnaire Dermatol 2008 217 2 101 106 10.1159/000128992 18451647

[B16] Porter J Beuf AH Lerner A Nordlund J Response to cosmetic disfigurement: patients with vitiligo Cutis 1987 39 6 493 494 3608575

[B17] Lim-Ong M Leveriza RM Ong BE Frez ML Comparison between narrow-band UVB with topical corticosteroid and narrow-band UVB with placebo in the treatment of vitiligo: a randomized controlled trial J Phillipine Dermatol Soc 2005 14 17 22

